# Pericoronary Adipose Tissue Attenuation Is Associated with High-Risk Plaque and Subsequent Acute Coronary Syndrome in Patients with Stable Coronary Artery Disease

**DOI:** 10.3390/cells10051143

**Published:** 2021-05-10

**Authors:** Jeremy Yuvaraj, Andrew Lin, Nitesh Nerlekar, Ravi K. Munnur, James D. Cameron, Damini Dey, Stephen J. Nicholls, Dennis T. L. Wong

**Affiliations:** 1 Monash Cardiovascular Research Centre, Monash University and MonashHeart, Clayton, VIC 3800, Australia; jeremy.yuvaraj@gmail.com (J.Y.); nitesh.nerlekar@gmail.com (N.N.); kiran.munnur@gmail.com (R.K.M.); james.cameron@monash.edu (J.D.C.); Stephen.Nicholls@monashhealth.org (S.J.N.); 2 Biomedical Imaging Research Institute, Cedars-Sinai Medical Center, Los Angeles, CA 90048, USA; andrewklin@gmail.com (A.L.); Damini.Dey@cshs.org (D.D.); 3 South Australian Health and Medical Research Institute, Adelaide, SA 5000, Australia

**Keywords:** coronary computed tomography angiography, atherosclerosis, coronary artery disease, high-risk plaque, acute coronary syndrome, pericoronary adipose tissue

## Abstract

Background: High-risk plaques (HRP) detected on coronary computed tomography angiography (CTA) confer an increased risk of acute coronary syndrome (ACS). Pericoronary adipose tissue attenuation (PCAT) is a novel biomarker of coronary inflammation. This study aimed to evaluate the association of PCAT with HRP and subsequent ACS development in patients with stable coronary artery disease (CAD). Methods: Patients with stable CAD who underwent coronary CTA from 2011 to 2016 and had available outcome data were included. We studied 41 patients with HRP propensity matched to 41 controls without HRP (60 ± 10 years, 67% males). PCAT was assessed using semi-automated software on a per-patient basis in the proximal right coronary artery (PCAT_RCA_) and a per-lesion basis (PCAT_Lesion_) around HRP in cases and the highest-grade stenosis lesions in controls. Results: PCAT_RCA_ and PCAT_Lesion_ were higher in HRP patients than controls (PCAT_RCA_: −80.7 ± 6.50 HU vs. −84.2 ± 8.09 HU, *p* = 0.03; PCAT_Lesion_: −79.6 ± 7.86 HU vs. −84.2 ± 10.3 HU, *p* = 0.04), and were also higher in men (PCAT_RCA_: −80.5 ± 7.03 HU vs. −86.1 ± 7.08 HU, *p* < 0.001; PCAT_Lesion_: −79.6 ± 9.06 HU vs. −85.2 ± 7.96 HU, *p* = 0.02). Median time to ACS was 1.9 years, within a median follow-up of 5.3 years. PCAT_RCA_ alone was higher in HRP patients who subsequently presented with ACS (−76.8 ± 5.69 HU vs. −82.0 ± 6.32 HU, *p* = 0.03). In time-dependent analysis, ACS was associated with HRP and PCAT_RCA_. Conclusions: PCAT attenuation is increased in stable CAD patients with HRP and is associated with subsequent ACS development. Further investigation is required to determine the clinical implications of these findings.

## 1. Introduction

Approximately 50% of acute coronary syndromes (ACS) arise from the rupture of non-occlusive coronary lesions [1,2]. Many of these lesions have features which predispose them to rupture and are termed high-risk plaques (HRP). Coronary computed tomography angiography (CTA) is a widely used non-invasive imaging modality which can reliably assess HRP features [3]. Importantly, the presence of HRP in patients with stable coronary artery disease (CAD) is associated with an increased prospective risk of ACS [4,5], highlighting the potential prognostic value of HRP detection on imaging. 

Chronic vascular inflammation plays a key role in atherogenesis and atherosclerotic plaque rupture. Pericoronary adipose tissue (PCAT) attenuation is a novel marker of coronary inflammation on coronary CTA, capturing changes in adipocyte size and lipid accumulation caused by inflammatory mediators from the vascular wall [6]. Measurement of PCAT around the proximal RCA represents a standardised and prognostically validated per-patient metric [7,8,9,10]. PCAT attenuation can also be quantified around identifiable coronary lesions as a dynamic marker of coronary inflammation [6,7,11]. While increased PCAT attenuation has been reported surrounding HRP [12,13,14], few studies have systematically evaluated the association between ACS and PCAT attenuation in patients with HRP. We sought to perform a per-patient and per-lesion level analysis of PCAT attenuation in stable CAD patients with HRP who develop subsequent ACS. 

## 2. Materials and Methods

### 2.1. Patients

A cohort of 1254 consecutive patients with available follow-up data, referred for coronary CTA between September 2008 and November 2017, was retrospectively collected. Stable CAD was defined as stable angina with no prior history of CAD. We excluded patients with poor scan quality as assessed by a five-point Likert scale [15] and those who underwent early revascularisation (<3 months). Patients were followed up for long-term outcomes until November 2017. Patients’ follow-up was a median of 5.3 years (IQR 5.1 years to 5.7 years). Two independent cardiologists blinded to patient data adjudicated the presence of HRP and ACS. Presence of HRP was determined via visual evaluation of coronary CTA images for HRP characteristics. ACS was defined as per the basis of the third universal definition of myocardial infarction with or without ST elevation (STEMI and NSTEMI, respectively) alongside troponin elevation, and unstable angina, assessed using a combination of medical records, blood results and findings on invasive angiography [16]. ACS, late unplanned revascularisation (>3 months from coronary CTA) and cardiac death were study endpoints. Three patients with ACS died from cardiac causes. This study was approved by the Monash Health Human Research Ethics Committee. 

### 2.2. Coronary CTA Protocol

Coronary CTA scans were performed using a 320-detector row scanner (Aquilion ONE; Canon Medical Systems Corporation, Otawara, Japan). Acquisitions were obtained during a single breath-hold with prospective ECG triggering, with use of beta-blockers for heart rate control at 60 bpm when required. Coronary CTA acquisition parameters were: detector configuration 320 × 0.5 mm; tube current 300–500 mA (depending on body mass index (BMI)); tube potential 100–120 kV; gantry rotation time 275 ms; and temporal resolution 175 ms. Iodinated contrast was administered (60–90 mL, 350 mg iodine per mL (Omnipaque)) at a rate of 5 mL/s. Image reconstruction was mediated by the adaptive iterative dose-reduction three-dimensional algorithm (AIDR3D, Canon Medical Systems) and FC43 reconstruction kernel to generate a 512 × 512 matrix with 0.5 mm slice thickness and 0.25 mm slice increments. 

### 2.3. Plaque Analysis

HRP was identified via visual assessment by two independent experts blinded to both patient demographics and clinical data (RM, DW). HRP was defined as the presence of at least two of the following features: positive remodelling (PR), low-attenuation plaque (LAP) or spotty calcification (SC). These features have been defined previously [4,17]. Briefly, PR was assessed by comparison of vessel diameter at the coronary lesion to diameter at a reference site proximal to the lesion. This ratio was quantified as a remodelling index (lesion diameter/reference diameter). If the lesion diameter was ≥10% larger than the reference segment, the lesion was defined as positively remodelled. LAP was defined as plaque attenuated at <30 HU. SC was defined on both multiplanar reconstructed images as calcification <3 mm, and on cross-sectional images as calcification covering <90° of the vessel arc. Obstructive CAD was defined on visual assessment as >50% diameter stenosis. 

Semi-automated software (Vitrea 6 Version 3.0 with SUREPlaque; Vital Images and Canon Medical Systems) was used to quantify total plaque volume, vessel and plaque diameter and plaque attenuation. Total plaque burden per lesion was calculated as: (total plaque volume/total vessel volume) × 100. CAD severity was also assessed by segment involvement score (SIS) and segment stenosis score (SSS). SIS refers to the total number of coronary segments with plaque, while SSS refers to the total stenosis score in each segment according to a five-point scale: 0, 0% stenosis (normal); 1, 1–24% stenosis (minor); 2, 25–49% stenosis (mild); 3, 50–69% stenosis (moderate); 4, 70–99% stenosis (severe) [18,19]. 

### 2.4. PCAT Analysis

PCAT was quantified in each patient around the proximal right coronary artery (PCAT_RCA_) and around specific identified lesions (PCAT_Lesion_)—culprit coronary lesions in patients with ACS, and the highest-grade stenosis lesions in patients with HRP without ACS and in controls, using semi-automated software (AutoPlaque v2.5, Cedars-Sinai Medical Centre, Los Angeles, CA, USA). Using this software, an experienced operator blinded to clinical data (JY) applied a centreline to the vessel containing the target plaque and demarcated adventitia from surrounding adipose tissue. Adipose tissue was defined as all voxels between −190 HU and −30 HU within a predefined volume of interest. This volume of interest included three-dimensional concentric layers extending outward from the operator-traced vessel wall to an extent of 3 mm, the typical diameter of the RCA [11]. PCAT was automatically quantified as the mean attenuation of all voxels within this volume. Analysis excluded all non-adipose tissue and vessel branches not delineated by the manually defined centreline and vessel wall.

PCAT_RCA_ is a standardised technique that provides information on the inflammatory status of the entire coronary tree and is measured in the proximal 10–50 mm of the RCA, a method which has been validated previously [6,7,8]. PCAT_Lesion_ was measured around target lesions, with the proximal and distal borders of the analysis region defined as the proximal and distal ends of the lesion [6,12,14].

### 2.5. Statistical Analysis

**Figure 1:**
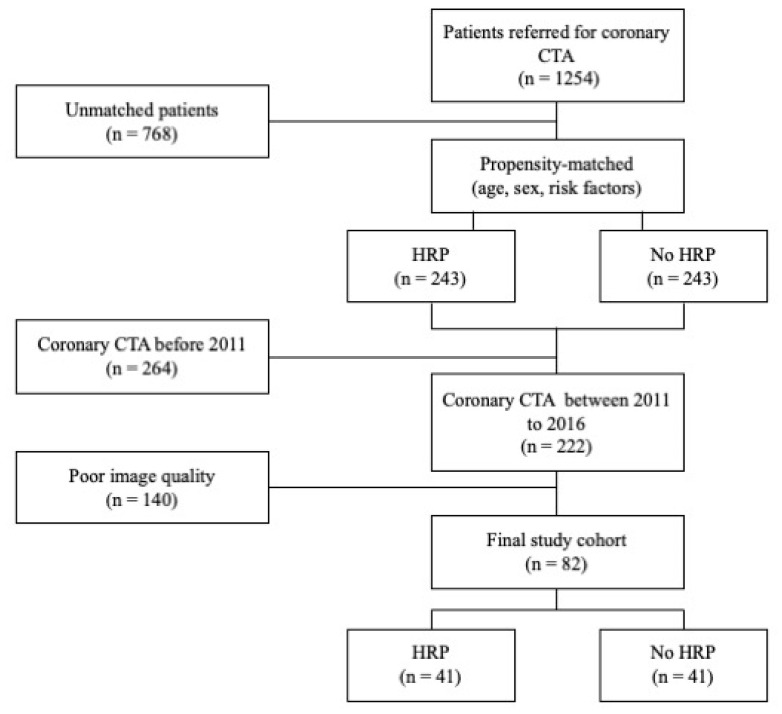
Patient selection and study design. Flow chart depicting selection of final study cohort, including patients with HRP matched to patients without HRP. CTA: computed tomography angiography; HRP: high-risk plaque.

The patient selection process is summarised in Figure 1. Of the 1254 patients, 243 patients with HRP were propensity-matched on a 1:1 basis to patients without HRP using a nearest-neighbour approach. Propensity-matching was performed on the basis of age, sex, hypertension, hypercholesterolaemia, diabetes mellitus, current smoking status and family history of ischaemic heart disease (IHD). Patients were excluded on the basis of poor scan quality or the use of dated scan parameters (*n* = 82, 16.9%). Scan quality was assessed via a five-point Likert score as described previously [15]. Differences in scan parameters within the initial cohort included iterative reconstruction algorithm, reconstruction kernel and the scanner itself. Only scans performed between 2011 and 2016 were included to maintain consistency in scan parameters between patients with HRP and controls. A total of 41 patients with HRP matched to 41 patients without HRP with coronary CTA being performed between 2011 and 2016 were investigated. 

Data were tested for normality using the Shapiro–Wilk test. Continuous variables are presented as mean ± standard deviation or median (interquartile range/IQR), as appropriate. Comparison of continuous variables between groups was assessed using the independent *t*-test. A Chi-square or Fisher’s exact test was used for categorical variables. The relationship between PCAT attenuation and age, sex and individual cardiovascular risk factors (hypertension, hypercholesterolaemia, diabetes mellitus, smoking, family history of IHD) was assessed using linear regression. Binary logistic regression was used to assess the relationship between the presence of HRP and PCAT_RCA_, correcting for the aforementioned covariates. Variables with a *p* < 0.20 on univariable analyses were included in multivariable models, and a two-sided *p*-value < 0.05 was considered statistically significant. Additionally, univariable Cox regression analysis was performed to determine the odds of ACS development against covariates found to be significant in logistic regression analysis. The censoring period for this analysis was defined as the date of coronary CTA to the time of ACS, or the date of follow-up in patients who did not present with ACS. All statistical analyses were performed using IBM SPSS Statistics (version 25).

## 3. Results

Baseline patient characteristics are summarised in Table 1. The final study population comprised 82 patients; mean age was 59.5 ± 9.7 years and 67% were males. Patients with HRP were well-matched to non-HRP patients in terms of age, sex and cardiovascular risk factors. Median time to ACS was 1.9 years (IQR 1.3 years to 4.2 years). ACS was more frequent in patients with HRP compared to controls, but this was not significant (24.4% (*n* = 10) vs. 7.3% (*n* = 3), *p* = 0.07).

### 3.1. Plaque and Vessel Characteristics

All patients with HRP had PR, with a median remodelling index of 1.5 (IQR, 1.31 to 1.73). Furthermore, LAP was present in 58.5% (*n* = 24) and SC was present in 53.7% (*n* = 22) of HRP patients. Obstructive CAD was observed in a small proportion of patients with HRP (36.6%, *n* = 15), and in one control patient (2.44%). Compared to patients without HRP, patients with HRP had a higher median SIS (4.5 (IQR 3 to 6) vs. 1.0 (IQR 0 to 4.5), *p* < 0.001) and SSS (5.5 (IQR 3 to 9.5) vs. 2.0 (IQR 0 to 6), *p* = 0.001 (Table 1)). 

### 3.2. Relationship between PCAT and HRP

**Figure 2:**
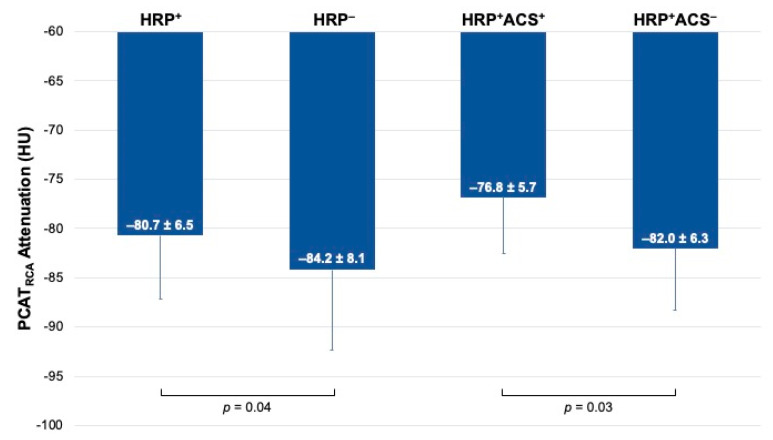
PCAT_RCA_ attenuation by presence of HRP and subsequent presentation with ACS. Bar graphs of per-patient differences in PCAT attenuation in patients with HRP (HRP^+^) and without HRP (HRP^−^), and in patients with HRP who developed ACS (HRP^+^ACS^+^) and patients with HRP who did not develop ACS (HRP^+^ACS^−^). ACS: acute coronary syndrome.

**Figure 3:**
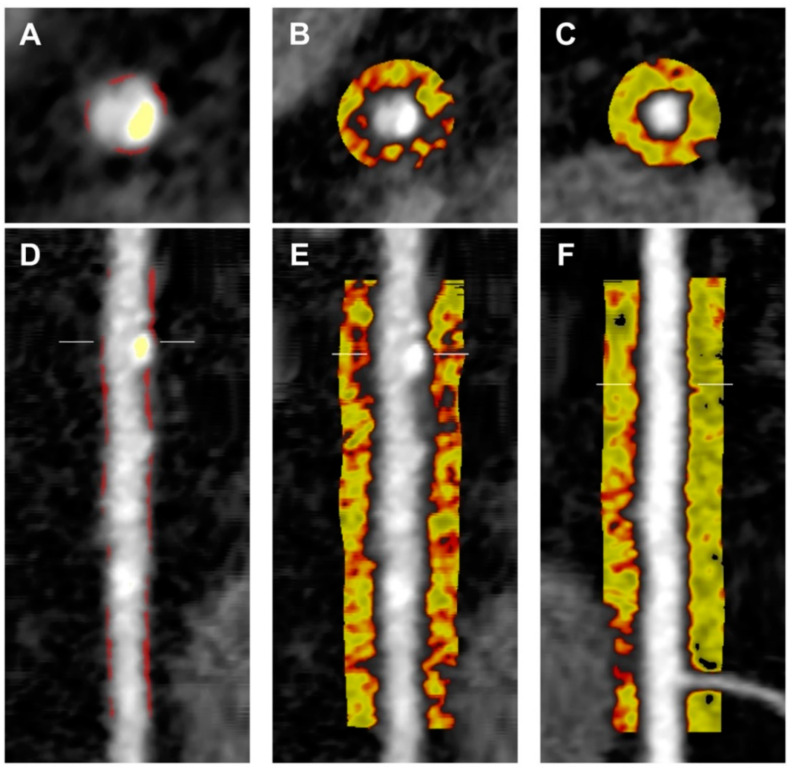
Case comparison of per-patient PCAT quantification. Comparison of the RCA of a patient with HRP versus a patient without HRP (control) as shown on coronary CTA. RCA in patient with HRP shown in cross-sectional (**A**) and longitudinal (**D**) views, with calcified plaque (yellow) and non-calcified plaque (red) highlighted. Representation of PCAT attenuation of patient with HRP (−75.6 HU) and control (−93.9 HU) shown in cross-sectional (**B**,**C**) and longitudinal (**E**,**F**) views. Colour map corresponds to CT attenuation in Hounsfield Units (HU) ranging from −190 HU (yellow) to −30 HU (red). Cross-sectional view in patient with HRP shown in panel (**B**) versus control shown in panel (**C**). Longitudinal view in patient with HRP shown in panel (**E**) versus control shown in panel (**F**). RCA: right coronary artery.

PCAT_RCA_ was significantly higher in patients with HRP compared to patients without HRP (−80.7 ± 6.5 HU vs. −84.2 ± 8.1 HU, *p* = 0.03 (Figure 2)). PCAT_Lesion_ was also higher in HRP lesions compared to the highest-grade stenosis lesions in controls (−79.6 ± 7.9 HU vs. −84.2 ± 10.3 HU, *p* = 0.04). PCAT_RCA_ was correlated with PCAT_Lesion_ (r = 0.513, *p* < 0.01), but there was no significant difference in PCAT_RCA_ and PCAT_Lesion_ within patients (−81.4 ± 6.8 HU vs. −81.4 ± 9.1 HU, *p* = 0.98). A visual representation of PCAT attenuation in a patient with HRP and a control patient is shown in Figure 3.

### 3.3. Relationship between PCAT and ACS

PCAT_RCA_ was higher in patients presenting with subsequent ACS compared to those without subsequent ACS (−78.03 HU ± 7.32 vs. −83.3 HU ± 7.29, *p* = 0.02). PCAT_Lesion_ was only numerically higher in patients presenting with subsequent ACS compared to those who did not present with ACS (−77.3 HU ± 8.82 vs. −82.4 HU ± 8.93, *p* = 0.07). 

PCAT_RCA_ was higher in HRP patients presenting with ACS than HRP patients who did not present with subsequent ACS (−76.8 HU ± 5.69 vs. −82.0 HU ± 6.32, *p* = 0.03 (Figure 2)). PCAT_Lesion_ was only numerically higher in patients with HRP who presented with ACS (−76.8 HU ± 9.44 vs. −80.5 HU ± 7.22, *p* = 0.2). 

### 3.4. Sex-Specific Differences in PCAT

**Figure 4:**
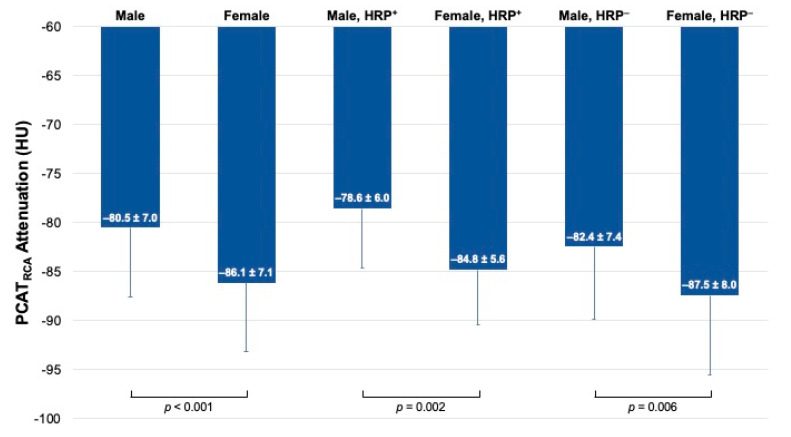
PCAT_RCA_ attenuation by gender and presence of HRP. Bar graphs of per-patient differences in PCAT attenuation in men (Male) and women (Female), men with HRP (Male, HRP^+^) and women with HRP (Female, HRP^+^), and men without HRP (Male, HRP^−^) and women without HRP (Female, HRP^−^). ACS: acute coronary syndrome.

Among all patients, both PCAT_RCA_ and PCAT_Lesion_ were significantly higher in men than in women (PCAT_RCA_: −80.5 ± 7.03 HU vs. −86.1 ± 7.08 HU, *p* < 0.001; PCAT_Lesion_: −79.6 ± 9.06 HU vs. −85.2 ± 7.96 HU, *p* = 0.02 (Figure 4)). Similar results were observed among patients with HRP (PCAT_RCA_: −78.6 ± 5.97 HU vs. −84.8 ± 5.58 HU, *p* = 0.002; PCAT_Lesion_: −77.7 ± 7.43 HU vs. −83.4 ± 7.52 HU, *p* = 0.03 (Figure 4)). 

### 3.5. Multivariable Regression Analysis

On multivariable linear regression analysis, sex, HRP and current smoking status were associated with PCAT_RCA_ (Table 2) after adjustment for ACS presentation. On multivariable logistic regression, PCAT_RCA_ was independently associated with the presence of HRP (Table 3).

### 3.6. Time-Dependent Analysis

On univariable Cox regression analysis, ACS was significantly associated with HRP presence (OR = 3.79 (95%CI 1.04 to 13.85), *p* = 0.04) and PCAT_RCA_ (OR = 1.09 (95%CI 1.01 to 1.18), *p* = 0.03), but not with gender (OR = 3.79 (95%CI 0.36 to 3.86), *p* = 0.78). Additionally, ACS was significantly associated with SSS (OR = 1.12 (95%CI 1.01, 1.24), *p* = 0.04) and the presence of obstructive CAD (OR = 4.80 (95%CI 1.60, 14.40), *p* < 0.01), but not by SIS (*p* = 0.27). No traditional cardiovascular risk factors were significantly associated with ACS (all *p* > 0.1). 

**Figure 5:**
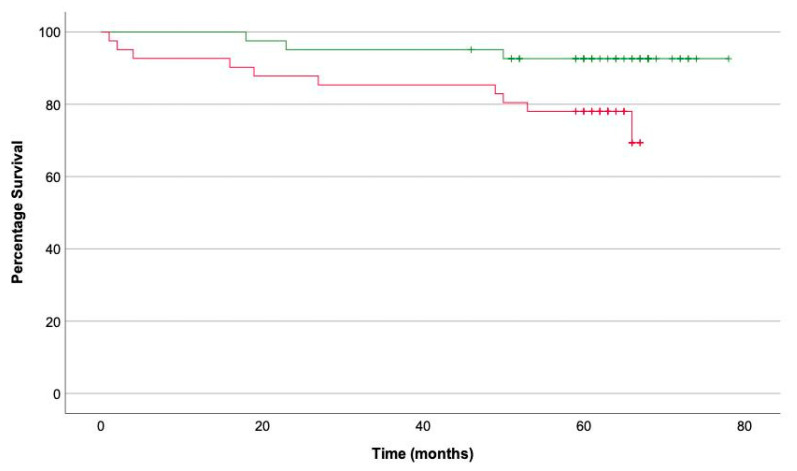
Survival curve of patients with HRP (red) versus patients without HRP (green).

On Kaplan–Meier analysis, patients with HRP harboured significantly greater risk of ACS than patients without HRP (log-rank *p* = 0.03 (Figure 5)). Patients were also stratified into high- and low-PCAT_RCA_ subgroups around a cutoff of the mean PCAT_RCA_ within the entire cohort (−82.4 HU ± 7.50). While patients with high-PCAT_RCA_ experienced lower percentage survival, this did not achieve significance (log-rank *p* = 0.08). 

## 4. Discussion

Our study evaluated the association between PCAT attenuation and ACS in patients with HRP features detected on coronary CTA. PCAT_RCA_, a well-validated metric of intracoronary inflammation, is increased in HRP-positive patients who go on to develop ACS compared to those who do not develop major coronary events. Furthermore, HRP presence and PCAT_RCA_ is associated with an increased rate of ACS. In addition, we also report sex-specific differences in PCAT attenuation, with increased attenuation being observed in men with HRP compared to women with HRP. The findings of this study highlight differentials in coronary inflammation in the context of ACS, particularly among a patient population at a greater risk of developing events. 

The relationship between inflammation and vulnerable atherosclerotic plaque is well-documented in the literature [2,20,21]. Observational studies have established a robust association between HRP and ACS [4,5,17]. Recent evidence arising from the SCOT-HEART study [22] demonstrates the strong predictive value of LAP for fatal and nonfatal myocardial infarction (MI) in a large study population. Moreover, patients within this cohort with the combination of both nonobstructive CAD and LAP burden >4% harboured a sixfold increased risk of MI, highlighting that vulnerable plaque morphology plays a critical role in the development of coronary events independent of plaque-related stenosis.

PCAT studies show that both pan-coronary and lesion-specific inflammation are associated with high-risk lesions in stable CAD [8,14], as well as major coronary events [7,12], indicating potential for PCAT attenuation to detect incipient CAD before the appearance of vulnerable plaque. We report that both HRP presence and PCAT_RCA_ were significantly associated with ACS development. Moreover, PCAT_RCA_ was higher in patients with HRP who then went on to develop ACS. A number of prior studies have evaluated the relationship between coronary inflammation and ACS in either per-patient [7] or per-lesion forms of PCAT assessment [12,14]. In per-patient analysis, Oikonomou et al. found that increased PCAT_RCA_ was predictive of MI in patients with stable CAD [7]. Likewise, Lin et al. found that per-patient PCAT attenuation was associated with AMI and was distinctly higher in these patients compared to stable CAD or control patients [10]. Studies evaluating per-lesion analysis demonstrated increased attenuation around culprit lesions in ACS [12,23] and MI [6,24]. Importantly, Gaibazzi et al. described increased per-vessel PCAT attenuation and HRP characteristics in patients with myocardial infarction with non-obstructive coronary artery disease (MINOCA) and Tako-Tsubo Syndrome (TTS) [24]. The largely non-obstructive nature of vulnerable plaque observed in our study is also evident in the literature [1,2,21,22] and further evidences that HRP features and plaque-specific inflammation may be additive to luminal stenosis in predicting event risk. Indeed, increased PCAT attenuation results in an increased prospective risk of cardiac mortality, among both patients with and without HRP [25]. Nevertheless, we found that only PCAT_RCA_ and not PCAT_Lesion_ were associated with ACS, though both metrics were correlated with one another. A possible explanation may reside in the significant range observed in the duration from coronary CTA to presentation with ACS in these patients; in some cases, PCAT_Lesion_ may have therefore reflected only a limited degree of inflammation surrounding plaques well before their development into culprit lesions. It is plausible that HRP may represent a series of structural changes appearing later in the inflammatory process which are more acutely related to vulnerability, as a number of studies demonstrate that HRP phenotypes associate with ACS occurring within a year of follow-up [4,26]. Moreover, PCAT_Lesion_ captures rapid changes in coronary inflammation that occur within weeks of MI [6], further underscoring the dynamic nature of plaque-specific inflammation. In contrast, increased PCAT_RCA_ demonstrates a level of inflammation throughout the coronary vasculature that may be present long before localisation to specific sites and culminates in HRP formation. Further studies investigating per-patient and per-lesion forms of PCAT assessment and ACS are required to fully elucidate the inflammatory changes occurring globally within the coronary vasculature and specific to culprit lesions. 

We also report significantly increased PCAT_RCA_ and PCAT_Lesion_ in men overall and specifically within groups with HRP. Increased vascular inflammation has been observed in men by means of elevated serum inflammatory biomarkers [27], but sex-specific differences in coronary inflammation remain unclear. Recent studies report increased PCAT attenuation in men compared to women [9,28], and the magnitude of HU difference in these studies was similar to our own findings, although we did not adjust for the influence of age or technical parameters. To the best of our knowledge, ours is the first study that reports increased coronary inflammation shown by both PCAT_RCA_ and PCAT_Lesion_ specifically in men with HRP. As stated previously, the association between HRP and cardiac events is well-documented in the literature [5], but the impact of sex on this association remains uncertain. In a propensity-matched study, Plank et al. found that men had a higher proportion of HRP features and MACE, but the presence of mixed plaque and non-calcified plaque was significantly more prevalent among women [29]. Importantly, numerous studies postulate that middle-aged women may indeed face a higher risk of MACE compared to men when obstructive CAD or HRP is present [29,30], while others demonstrate higher incidence of ACS among men despite a similar prevalence of HRP [31]. While it is clear that sex-specific differences may exist in plaque morphology, elucidating potential differentials in coronary inflammation in the context of vulnerable coronary disease may offer a level of clarity in this area and indicate if there is indeed a heightened risk of major coronary events faced by either sex. The low number of events in our study limited the capacity to further stratify associations between PCAT attenuation and ACS by sex. However, the finding of increased PCAT_RCA_ in men with HRP, coupled with the association of PCAT_RCA_ with ACS, calls for further investigation into this area to explore the potentially disproportionate risk of major coronary events that men may face with or without HRP. 

As atherosclerosis is recognised as a disease of chronic inflammation, the advent of PCAT attenuation as an indirect metric of coronary inflammation provides a useful diagnostic and prognostic tool in evaluating inflammatory risk. This study examines current forms of PCAT assessment in the context of stable CAD patients with HRP, finding that PCAT attenuation is able to detect not only the presence of HRP but the subsequent development of ACS. PCAT therein possesses diagnostic value, enabling indirect assessment of inflammatory risk in individuals with suspected CAD. Incorporation of PCAT assessment into coronary CTA-based risk stratification has been espoused previously [7], and may enhance primary prevention and potential delineation of inflammatory risk prior to plaque formation. Moreover, higher degrees of coronary inflammation shown by increased PCAT would allow for timelier and proactive clinical intervention. 

While these findings are novel, this was a small, retrospective, single-centre study. This consequentially limited the number of patients with ACS studied in this cohort, which may have influenced our multivariable analysis of ACS and PCAT_RCA_, and restricted us to only univariable time-dependent analysis of these variables. Development of ACS in patients with CAD would be best studied by large longitudinal studies, though we do report increased PCAT_RCA_ even among a small proportion of ACS cases. We performed both PCAT_RCA_ and PCAT_Lesion_ assessment, but the latter may be hampered by factors pertaining to luminal attenuation in the left anterior descending and left circumflex arteries, which decline alongside decreases in luminal diameter and contrast enhancement from ostial to distal segments [32]. Moreover, partial volume averaging due to the spatial resolution limitations of coronary CTA may affect attenuation measurements in pericoronary fat. Finally, data on medical therapy, such as statins and antiplatelet therapy, were not collected for this cohort and were therefore not included in adjusted analyses.

## 5. Conclusions

PCAT_RCA_ and PCAT_Lesion_ are increased in stable CAD patients with HRP compared to those without HRP. PCAT_RCA_ attenuation is higher in patients with HRP who develop ACS and predicts ACS in conjunction with the presence of HRP. PCAT attenuation is higher in men compared to women with HRP. This non-invasive imaging biomarker may identify patients at greater risk of future events, but clinical application of these measures requires further investigation.

## Figures and Tables

**Table 1:** Patient and plaque characteristics.

	HRP (*n* = 41)	No HRP (*n* = 41)	*p*-Value
**Cardiovascular risk factors**			
Age, years	59.8 (1.45)	59.2 (1.60)	0.77
Male sex	27 (65.9)	27 (65.9)	1.0
Hypertension	16 (39.0)	15 (36.6)	0.82
Hypercholesterolaemia	22 (53.7)	16 (39.0)	0.18
Diabetes mellitus	6 (14.6)	3 (7.3)	0.48
Smoker	7 (17.1)	4 (9.8)	0.52
Family history of IHD	22 (53.7)	22 (53.7)	1.0
Obesity	3 (7.30)	2 (4.90)	1.0
ACS	10 (24.4)	3 (7.30)	0.07
**Vessel localisation**			
LAD	26 (63.4)	34 (82.9)	
LCx	4 (9.76)	2 (4.88)	
RCA	11 (26.8)	5 (12.2)	
**Plaque characteristics**			
PR	41 (100)	-	
LAP	24 (58.5)	-	
SC	22 (53.7)	-	
Remodelling index *	1.5 (1.31, 1.73)	-	
Obstructive CAD	15 (36.6)	1 (2.44)	<0.001
Total plaque volume	55.6 (6.93)	-	
Total plaque burden	152 (64.6)	-	
CCTA segment scores *			
Segment involvement score	4.5 (3, 6)	1.0 (0, 4.5)	<0.001
Segment stenosis score	5.5 (3, 9.5)	2.0 (0, 6)	0.001

Values are expressed as *n* (%), or as mean (SD). * Median (IQR).

**Table 2:** Univariable and multivariable linear regression analyses of covariates and PCAT_RCA_.

	Univariable			Multivariable		
Variable	Beta	95%CI	*p*-value	Beta	95%CI	*p*-value
Age	−0.032	−0.204, 0.140	0.712			
Male sex	5.631	2.367, 8.895	0.001	5.085	1.986, 8.814	0.002
Presence of HRP	3.437	0.210, 6.663	0.037	3.017	0.087, 5.947	0.044
Presentation with ACS	5.238	0.851, 9.626	0.020	0.156	−0.970, 7.338	0.131
Hypertension	0.469	−2.948, 3.887	0.785			
Hypercholesterolaemia	1.232	−2.082, 4.545	0.462			
Diabetes	−0.174	−5.478, 5.130	0.948			
Smoker	7.146	2.548, 11.744	0.003	5.733	1.396, 10.070	0.010
Family history of IHD	1.366	−1.945, 4.677	0.414			
Obesity	−2.505	−9.412, 4.401	0.472			

**Table 3:** Univariable and multivariable logistic regression analyses of covariates and HRP.

	Univariable			Multivariable		
Variable	OR	95%CI	*p*-value	OR	95%CI	*p*-value
Age	1.007	0.963, 1.053	0.766			
Male sex	1.0	0.401, 2.491	1.0			
PCAT_RCA_	1.066	1.003, 1.134	0.041	1.064	1.000, 1.132	0.049
Hypertension	1.109	0.454, 2.710	0.820			
Hypercholesterolaemia	1.809	0.752, 4.352	0.186	1.723	0.701, 4.238	0.236
Diabetes	2.171	0.504, 9.350	0.298			
Smoker	1.904	0.512, 7.085	0.337			
Family history of IHD	1.0	0.420, 2.382	1.0			
Obesity	1.539	0.243, 9.733	0.647			

## Data Availability

No new data were created or analysed in this study. Data sharing is not applicable to this article.
